# Now more than ever: Mpox renews the call for local pharmaceutical production for Africa’s health security

**DOI:** 10.4102/jphia.v15i1.783

**Published:** 2024-09-26

**Authors:** Nicaise Ndembi, Morẹ́nikẹ́ O. Foláyan

**Affiliations:** 1 Africa Centres for Disease Control and Prevention (Africa CDC), Addis Ababa, Ethiopia; 2 Department of Child Dental Health, Obafemi Awolowo University, Ile-Ife, Nigeria

The first 10 000 doses of the mpox vaccine only arrived in Africa during the week of 26 August 2024^1^ and was followed by 200 000 doses in the Democratic Republic of Congo (DRC) – 2 years after mpox was first recognised as a public health challenge. In 2022, when the World Health Organization (WHO) declared mpox a public health emergency of international concern,^2^ Africa had no access to the vaccine. Even after the global public health emergency was declared over, cases continued to rise in the DRC, the epicentre of the outbreak. Despite the high number of cases in the DRC and the challenging conditions that hampered outbreak control, the first doses of the vaccine in Africa were received by Nigeria, not the DRC. Nigeria and DRC now join the other 70 countries outside Africa that have had access to the vaccine.

Limited access to the mpox vaccine is largely because of its cost. A single dose of the mpox vaccine, produced by Denmark’s Bavarian Nordic, costs around $100, and individuals require two doses. It is estimated that 10 million doses may be needed across Africa.^3^ The emergency use listing of mpox vaccines by manufacturers, initiated by the WHO in August 2024, is a process that comes many years too late. Since 2023, more than 1300 deaths have been reported in the DRC.^4^

The mpox outbreaks across Africa have once again underscored the critical need for local pharmaceutical manufacturing to ensure continental health security. The ongoing challenges in accessing vaccines for mpox mirror the difficulties faced with accessing vaccines during the coronavirus disease 2019 (COVID-19) pandemic. Difficulty with accessing vaccines, both the mpox and COVID-19 vaccines, are linked with cost. Cost is also a limiting factor for accessing human papillomavirus (HPV) vaccine. Human papillomavirus vaccines, crucial for preventing cervical cancer, have been inconsistently available in many African countries.^5^

Despite global efforts to improve vaccine distribution, Africa continues to face significant delays and inequities in access, largely because of its heavy reliance on external sources for pharmaceutical products – including vaccines, medicines, personal protective equipment and other medical supplies. The continent currently imports 99% of its vaccines and 95% of its medicines. This overwhelming dependence on external agencies leaves the health of 1.3 billion Africans uncertain, especially during global health crises.^6^ The absence of local production facilities in many African countries exposes them to the whims of global supply chains, which often prioritise wealthier nations, thereby highlighting systemic vulnerabilities in Africa’s health infrastructure

The need for local manufacturing of pharmaceuticals, including vaccines, had long been highlighted as essential for achieving continental health security in Africa.^7^ Developing local production capabilities would allow African nations to respond more swiftly to health emergencies, and it would support economic growth by creating jobs and fostering innovation within the pharmaceutical sector. Countries like South Africa, Egypt and Senegal have made strides in building their vaccine production capacities, but these efforts need to be scaled across the continent.

Building a robust local manufacturing sector requires collaboration and a coordinated approach between governments, private sector stakeholders and international partners to address technical, regulatory and financial barriers. This collaboration is vital in pooling resources and expertise, reducing duplication of efforts and ensuring that all African countries benefit from advancements in pharmaceutical manufacturing. It can lead to the creation of Regional Pharmaceutical Manufacturing Hubs as a transformative step towards achieving continental health security.

The African Union, through the Africa Centres for Disease Control and Prevention, has recognised the importance of pharmaceutical sovereignty and has called for increased investment in local manufacturing. The establishment of the African Medicines Agency is a step in the right direction, aiming to harmonise regulatory processes across countries and accelerate the approval of locally produced pharmaceuticals.

The mpox outbreaks have reignited the urgent call for Africa to prioritise local pharmaceutical manufacturing as a cornerstone of its health security strategy. The challenges faced in accessing vaccines for mpox during the current outbreak serve as a stark reminder that reliance on external sources is not sustainable for Africa’s health needs. Now more than ever, African nations must invest in local manufacturing to safeguard the health of their populations and build a more resilient and self-sufficient healthcare system. The time to act is now to ensure that Africa is better prepared for future health emergencies and thereby effectively contribute to global health security.
